# Composition and Activities of *Carpesium macrocephalum* Franch. & Sav. Essential Oils

**DOI:** 10.3390/molecules29194658

**Published:** 2024-09-30

**Authors:** Anna Wajs-Bonikowska, Janusz Malarz, Łukasz Szoka, Paweł Kwiatkowski, Anna Stojakowska

**Affiliations:** 1 Institute of Natural Products and Cosmetics, Faculty of Biotechnology and Food Sciences, Łódź University of Technology, Stefanowskiego Street 2/22, 90-537 Lodz, Poland; 2 Maj Institute of Pharmacology, Polish Academy of Sciences, Smętna Street 12, 31-343 Krakow, Poland; malarzj@if-pan.krakow.pl; 3 Department of Medicinal Chemistry, Faculty of Pharmacy with the Division of Laboratory Medicine, Medical University of Bialystok, Mickiewicza Street 2D, 15-222 Bialystok, Poland; lukasz.szoka@umb.edu.pl; 4 Department of Diagnostic Immunology, Pomeranian Medical University in Szczecin, Powstańców Wielkopolskich Street 72, 70-111 Szczecin, Poland; pawel.kwiatkowski@pum.edu.pl

**Keywords:** alantolactone, cytotoxicity, linalool, melanoma, nerol, *Staphylococcus aureus*, thymol derivatives, thymol methyl ether

## Abstract

*Carpesium macrocephalum*, a species native to China, Korea, Japan, and Russia, has been used medicinally in the countries of its origin. Though mono- and sesquiterpenoids are known constituents of *C. macrocephalum*, the complete analysis of essential oils produced by the roots and aerial parts of the plant has not been published until now. The present study discloses considerable differences in the composition and cytotoxic activity of essential oils distilled from roots and shoots of *C. macrocephalum*. The GC-MS-FID analyses have led to the identification of 131 compounds in all, of which 114 were found in aerial parts and 110 in the roots of the plants. The essential oil distilled from shoots contained a mixture of nerol and thymol methyl ether (c. 26%), neryl isobutyrate (c. 12%) and linalool (c. 9%) as major constituents, whereas alantolactone (c. 29%), thymol methyl ether (c. 7%) and 2,5-dimethoxy-*p*-cymene (thymohydroquinone dimethyl ether, c. 7%) predominated in the essential oil obtained from the roots. The oils demonstrated weak antibacterial activity against *Staphylococcus aureus* and, at concentrations up to 2.08 mg/mL (oil from the aerial parts) and up to 3.38 mg/mL (oil from roots), were inactive against Gram-negative bacteria. The essential oil from the roots of the plant demonstrated strong but not selective cytotoxic activity.

## 1. Introduction

*Carpesium macrocephalum* Franch. & Sav. (synonym: *Carpesium eximium* C. Winkl., Inuleae-Inulinae, Asteraceae) is a perennial plant that inhabits deciduous or mixed forests in China, Japan, Korea, and Russia. The herb, ca. 1 m tall, has a flexuous crisp-pubescent stem and terminal capitula (25–35 mm in diameter), composed of yellow, tubular disc and marginal florets, which are surrounded by linear or lanceolate bracts [1]. Traditionally, in China and Korea, the herb has been used as an analgetic, antihemostatic, antipyretic, and vermifuge agent, as well as in order to suppress inflammatory conditions [2,3].

Phytochemical investigations of plants from the genus *Carpesium* led to the isolation of numerous biologically active compounds [2,4,5], mostly representatives of mono- and sesquiterpenoids. *C. macrocephalum* remains one of the less investigated medicinally used species of the genus. Konovalova and coworkers isolated sesquiterpene lactones: carabrone, telekin, ivalin, and carpesin from the aerial parts of *C. eximium* plants, collected in the south of the Maritime Territory (Russia) [6]. A new eudesmanolide from the aerial parts of *C. macrocephalum* and two eudesmanolide glucosides from the seeds of the plant were described by Yang et al. [7,8]. The dried whole plants of Korean origin yielded three guaianolides: 4*β*,10*β*-dihydroxy-1*α*(H),5*α*(H)-guai-11(13)-en-8*α*,12-olide, 4*α*,10*α*-dihydroxy-1*β*(H),5*β*(H)-guai-11(13)-en-8*α*,12-olide and 4*β*,10*β*-dihydroxy-5*α*(H)-guaia-1,11(13)-dien-8*α*,12-olide [9]. Detailed analysis of the methanol extract from seeds of *C. macrocephalum,* except for the two eudesmanolide glucosides described earlier, revealed the presence of eight eudesmanolides, carabrone, carabrol, one coumarin (scopoletin), 3,5,7,4′-tetrahydroxydihydroflavonol, *β*-sitosterol and daucosterol [10]. From the dried aerial parts of the plants collected in Gansu province (China), two new eudesmanolides together with telekin, 11*α*,13-dihydrotelekin, 2*α*,5*α*-dihydroxy-11*α*(H)-eudesma-4(15)-en-12,8*β*-olide, ivalin, 11*α*,13-dihydroivalin, carabrone, carabrol, scopoletin, *β*-sitosterol, and daucosterol were isolated [11]. The whole *C. macrocephalum* plants, harvested in Korea, provided five sesquiterpene lactones (carabron, carabrol, tomentosin, ivalin, 4*H*-tomentosin) and three other low-molecular-weight terpenoids (vomifoliol, loliolide, citrusin C) [12]. Five monoterpenoid thymol derivatives, including (*Z*)-10-isobutyryloxy-9-chloro-8,9-dihydrothymol, and three new sesquiterpene lactone dimers (carpedilactones E-G) were isolated from the whole plants of Chinese origin [13,14]. In addition to the previously identified compounds, a new xanthanolide (4-(2-methylbutyryl)-4*H*-tomentosin), *α*-costic acid, 5*α*-epoxyalantolactone, and 4*α*,5*α*-epoxy-10*α*,14*H*-1-*epi*-inuviscolide were found in a methanol extract from *C. macrocephalum*. Some of the described sesquiterpene lactones inhibited *Candida albicans* biofilm formation or the yeast-to-hyphae transition in the fungal cells [15].

A hydroalcoholic extract from *C. macrocephalum* demonstrated inhibitory activity towards histamine release from rat mast cells and against nitric oxide (NO) production by the activated RAW 264.7 murine macrophage cell line [16]. A methanol extract from the Korean plants inhibited lipopolysaccharide (LPS)-induced NO and PGE_2_ (prostaglandin E_2_) production in murine macrophages by suppression of inducible nitric oxide synthase (iNOS) and cyclooxygenase-2 (COX-2) activity due to suppression of mRNA and protein expression [3]. Moreover, the extract attenuated pro-inflammatory cytokines production inhibited protein kinase B (Akt) and the nuclear transcription factor NF-κB activation, as well as expression of signal transducer and activator of transcription proteins (STATs) and diminished vascular permeability in mice. The suppression of the NO production and other anti-inflammatory effects of the extract may be explained, at least in part, by the presence of sesquiterpene lactones [17].

Essential oils (EOs) produced by plants are aromatic mixtures of volatile, low-molecular-weight compounds of a hydrophobic nature. Terpenoids and hydrocarbons are their principal components. Apart from their use in perfumery, pharmaceutical, cosmetic, and food industries (food aromatization), EOs play a significant role in the ecological relationships of plants within their environment (allelopathy, deterrence, attraction of pollinators). Antimicrobial activities of EOs, as well as their effects on the human nervous system, are well documented [18,19,20,21]. Their cytostatic and anti-inflammatory effects have been also described [22,23,24]. Numerous mono- and sesquiterpenoids isolated from *Carpesium* spp., including those found in *C. macrocephalum*, demonstrated antibacterial and cytotoxic activities against different microbial and cancer cell lines [2]. As the EOs are mixtures of low-molecular-weight, volatile plant metabolites (including mono- and sesquiterpenoids), it may be expected that the EOs distilled from *C. macrocephalum* may have antibacterial or cytotoxic effects too. Our previous studies on compositions and activity of the EOs from closely taxonomically related species of the Inuleae-Inulinae: *Telekia speciosa* (Schreb.) Baumg., *Carpesium divaricatum* Sieb. & Zucc., and *Carpesium cernuum* L. disclosed the presence of numerous monoterpenoid thymol derivatives in the examined oils (especially in the EOs from the plant roots) [25,26,27]. The EOs distilled from *T. speciosa* and *C. cernuum* demonstrated antibacterial activity against both Gram-positive and Gram-negative bacteria and cytotoxicity against several human melanoma cell lines in vitro. The aim of the present study was to reveal the chemical composition of the previously unanalyzed EOs from roots and aerial parts of *C. macrocephalum* and to assess their antibacterial and cytotoxic effects.

## 2. Results

### 2.1. Chemical Composition of C. macrocephalum Essential Oils

The aerial parts of *C. macrocephalum* contained 0.08% (*w*/*w*) of a yellowish, fragrant essential oil (APEO). The 114 compounds that were identified as components of the APEO (all listed in Table 1) accounted for 96% of the oil. The primary constituents of the APEO were a mixture (9:1) of nerol and thymol methyl ether (**32** and **33**; c. 26%), neryl isobutyrate (**76**; c. 12%) and linalool (**18**; c. 9%). Thymohydroqinone dimethyl ether (2,5-dimethoxy-*p*-cymene; **58**) together with other thymol derivatives like thymol methyl ether (**33**), 6-methoxythymyl isobutyrate (**109**), **40**, **70**, **112**, **113**, **122**, **123**, and **134**, and sesquiterpene lactones of eudesmane type (alantolactone, **121**; isolantolactone, **125** and dihydroisoalantolactone, **120**) constituted c. 12.9% and c. 1% of the APEO, respectively. Five components of the APEO (**94**, **97**, **98**, **108**, and **118**) remained unidentified.

The distillation yield for the EO from roots of *C. macrocephalum* (REO) was 0.13% (*w*/*w*). Alantolactone (**121**) was the most abundant constituent of the REO (c. 29.3%). The remaining eudesmanolides (isoalantolactone, **125**; alloalantolactone, **126**; isoalantodiene, **128**) and thymol derivatives (**33**, **40**, **58**, **70**, **71**, **109**, **112**, **113**, **122**, **123**, **131**, **132**, and **134**) accounted for c. 5.2% and c. 20.7% of REO, respectively. The 110 identified components of REO made up c. 96.7% of the oil. Structures of four constituents (**94**, **98**, **108**, and **129**) were not resolved.

### 2.2. Antibacterial and Cytotoxic Activities of C. macrocephalum Essential Oils

At the concentration range tested (1.02–2080 μg/mL, APEO; 1.65–3380 μg/mL, REO), both APEO and REO were inactive against Gram-negative bacteria (*Escherichia coli* ATCC 25922 and *Pseudomonas aeruginosa* ATCC 27853) and modestly inhibited growth of methicillin-resistant (ATCC 43300) and methicillin-susceptible (ATCC 29213) strains of *Staphylococcus aureus* (APEO, MIC = 2.08 mg/mL; REO, MIC = 3.38 mg/mL). The results of the antibacterial activity assessment and reference values, estimated for thymol and gentamycin sulfate, are summarized in Table 2.

**Figure 1:**
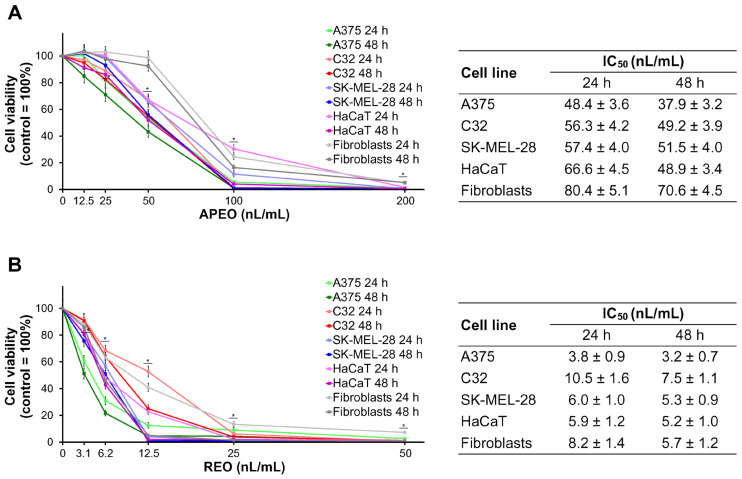
Cell viability after the treatment of human melanoma and normal skin cell lines with the essential oils from *Carpesium macrocephalum* for 24 h and 48 h: APEO (**A**) and REO (**B**). Data are reported as mean ± SD from three independent experiments. * *p* < 0.05 compared to control group.

As shown in Figure 1, REO demonstrated much higher cytotoxic activity towards the investigated human cell lines in vitro than APEO. Viabilities of melanoma cells (A375, C32, and SK-MEL-28), dermal keratinocytes (HaCaT), and fibroblasts were assessed after the treatment with APEO and REO for 24 h and 48 h. APEO (Figure 1A) was cytotoxic in all tested cell lines at a concentration of 50 nL/mL and above. Its IC_50_ values, after 24 h of the treatment, ranged from 48.4 nL/mL in A375 cells to 80.4 nL/mL in fibroblasts. Extension of the exposure time to 48 h resulted in a further decrease in the cell viability (from 10% in SK-MEL-28 cells to 27% in HaCaT keratinocytes). REO (Figure 1B) was cytotoxic in all tested cell lines at a concentration of 6.2 nL/mL and above. Its IC_50_ values, after 24 h of the treatment, were from 5.3 times (C32 cells) to 12.7 times (A375 cells) lower when compared with those for APEO and ranged from 3.8 nL/mL in A375 cells to 10.5 nL/mL in C32 cells. Similarly to the effects observed in the cells exposed to APEO, prolonged time of incubation (48 h) with REO resulted in a moderate reduction in cell viability (from 12% in SK-MEL-28 cells and HaCaT keratinocytes to 29% in C32 cells).

**Figure 2:**
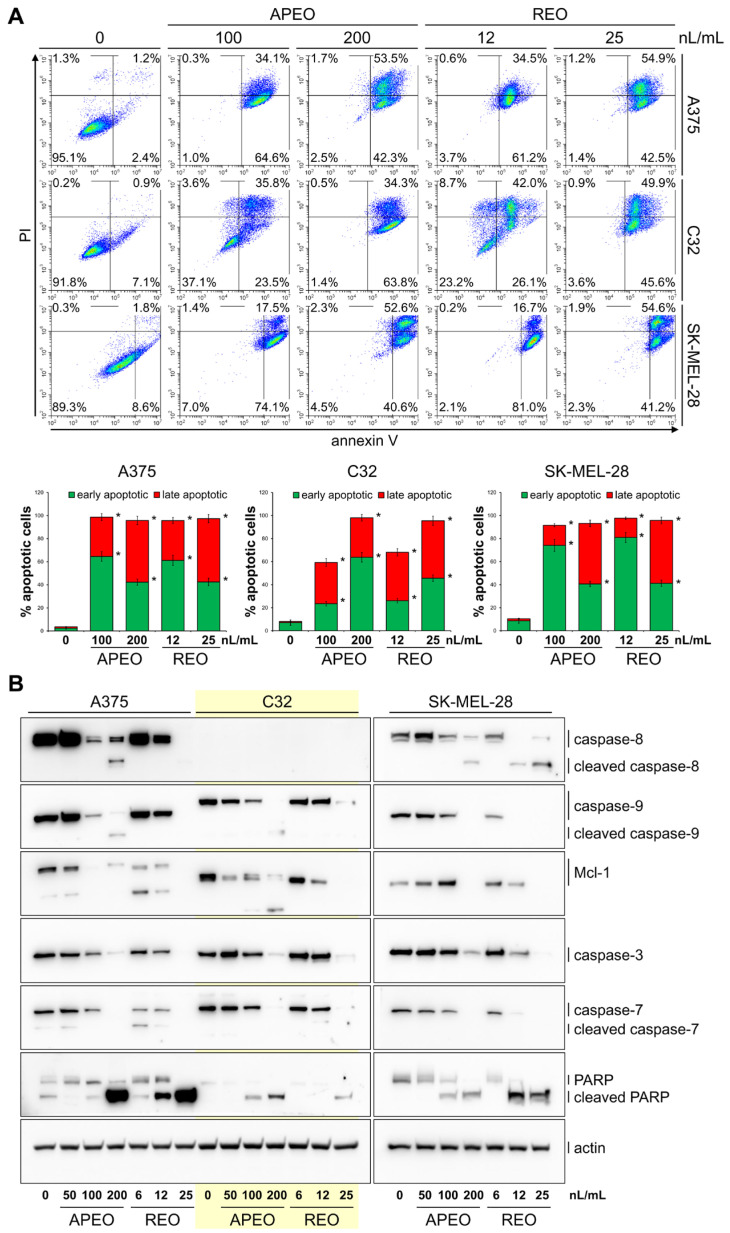
Apoptosis of melanoma cells after treatment with APEO and REO for 48 h (**A**). Western blot analysis of caspase-8, caspase-9, Mcl-1, caspase-3, caspase-7, and PARP in melanoma cells after the treatment with APEO and REO for 48 h. Actin served as a control for protein loading (**B**). Data are presented as mean ± SD from three independent experiments. * *p* < 0.05 compared to control group.

Analysis of apoptosis in melanoma cells (Figure 2A) treated with APEO at a concentration of 100 nL/mL showed a significant increase in the percentage of apoptotic cells: 59.3% in the C32 cell line, 91.6% in SK-MEL-28 cells and 98.7% in A375 cells. The REO exerted a much stronger effect and, at a concentration of 12 nL/mL, caused an increase in the percentages of apoptotic cells in line C32 (68.1%), in A375 cells (95.7%), and in SK-MEL-28 cells (97.7%). The levels of the key mediators of apoptosis in melanoma cells treated with APEO and REO for 48 h were evaluated by western blotting. As shown in Figure 2B, treatment of the cells with both EOs resulted in the downregulation of initiator caspase zymogen levels and their cleavage to active forms. Expression of (pro)caspase-8 in the cell line C32 was undetectable. The level of antiapoptotic Bcl-2 family member Mcl-1 gradually decreased with the increasing concentrations of APEO and REO. These changes were accompanied by the reduction in the levels of zymogens of executioner caspase-3 and caspase-7 and with the processing of poly(ADP-ribose) polymerase (PARP) into its cleavage product (cPARP).

## 3. Discussion

The genus *Carpesium*, according to the current taxonomic viewpoint based on the results of DNA sequencing, is closely related to *Telekia speciosa* (Schreb.) Baumg. and the genus *Inula* in its current form (including *I. helenium* L.), the group of large herbs with radiate capitula and resin canals in the stem [28,29]. The composition and antimicrobial activity of EOs from roots of *I. helenium* and different organs of *T. speciosa* have been repeatedly studied [30,31,32,33]. Even recently, a paper on the cytotoxic activity of the EO obtained from flowers of *T. speciosa* and the antibacterial activities of the EOs accumulated by leaves, roots, and flowers of the plant has been published [27]. Much less is known about the composition and biological activity of EOs from *Carpesium* spp. Most data concern *C. abrotanoides*, the species that has been used as a TCM (traditional Chinese medicine) for a long time in China. The data on the composition of essential oils from *C. abrotanoides* L., however, are not congruent. Kameoka et al. [34], from a “commercial herb sample” of *C. abrotanoides*, distilled the EO that contained *β*-bisabolene as the dominant constituent (c. 24.7%). The EOs obtained from the whole plants of *C. abrotanoides* by Wang et al. [35] contained eudesma-5,11(13)-dien-8,12-olide (alantolactone or its stereoisomer; c. 21.9%) and caryophyllene oxide (c. 13.0%), previously undetected by Kameoka et al., as major components. *β*-Bisabolene made up about 7% of the EO. Haris et al. [36] found caryophyllene (c. 24.3%), trans-nerolidol (c. 12.0%), geranyl isobutyrate (c. 10.6%), and δ-cadinene (c. 8.8%) in the EO from fresh aerial parts of *C. abrotanoides*. The EO investigated by Wang et al. [35] demonstrated moderate activity against human hepatocellular carcinoma (Hep G2) cells in vitro (IC_50_ = 22.59 ± 6.51 μg/mL after 48 h treatment) by inducing apoptosis via the mitochondrial pathway. The EO obtained from fresh aerial parts of *C. abrotanoides* showed some effectiveness against *Aedes aegypti*, a vector of the dengue virus [36].

α-Pinene, the major constituent in the EOs from aerial parts of *C. divaricatum* and *C. cernuum* (c. 40.2% and 34.7%, respectively) [25,26], was not detected in the EOs distilled from *C. macrocephalum*. Nerol and neryl isobutyrate that dominated in the *C. macrocephalum* APEO occurred as major constituents in the EO from aerial parts of *C. divaricatum* (c. 3.7% and 3.2%) and were present in smaller quantities in the EOs from *C. cernuum.* Thymohydroquinone dimethyl ether was found in the APEO from *C. macrocephalum* (c. 7.9%) and in the EOs from aerial parts of *C. cernuum* and *C. divaricatum* (c. 11.6% and 2.1%, respectively). Linalool, which constituted c. 9.3% of *C. macrocephalum* APEO, made up c. 4.3% and 2.1% of the essential oils from aerial parts of *C. cernuum* and *C. divaricatum*, respectively. Alantolactone/eudesma-5,11(13)-dien-8,12-olide was the only sesquiterpene lactone of eudesmane type that was identified in EOs from aerial parts of *C. cernuum* and *C. divaricatum*. Unlike in the EO from whole plants of *C. abrotanoides* [35], alantolactone was a minor constituent of *C. cernuum* and *C. divaricatum* EOs (c. 0.1%). *C. macrocephalum* APEO contained at least three eudesmanolides (alantolactone, isoalantolactone, and dihydroisoalantolactone), albeit in small quantities (c. 0.1–0.7%).

In contrast to the roots of *C. cernuum* and *C. divaricatum*, the roots of *C. macrocephalum* contained an EO that was rich in alantolactone (c. 29.3%). The other eudesmanolides identified in the REO were alloalantolactone (c. 4.4%), isoalantolactone (c. 0.7%), and isoalantodiene (c. 0.1%). High contents of eudesmanolides are characteristic of the EOs from roots of *T. speciosa* (c. 46.2–83.4% of isoalantolactone and c. 0.2–2.6% of alantolactone) [27,32,33] and *I. helenium* (alantolactone, c. 42.3–65.8% and isoalantolactone, c. 25.5–37.3%) [30]. Thymohydroquinone dimethyl ether that constituted c. 54.8% of the EO from *C. cernuum* roots made up c. 2.7% of the EO from roots of *C. divaricatum* and c. 6.6% of *C. macrocephalum* REO. The most abundant (c. 29.2%) constituent of the EO from roots of *C. divaricatum*, 10-isobutyryloxy-8,9-epoxythymyl isobutyrate, was identified in the EOs from roots of *T. speciosa* (c. 1.4–2.9%), *C. cernuum* (c. 5.1%) and *C. macrocephalum* REO (c. 2.2%) [25,26,27,32,33]. The remaining major constituents of REO were as follows: thymol methyl ether (c. 7.1%), isoshyobunone (c. 5.6%), modheph-2-ene (c. 4.6%) and caryophyllene epoxide (c. 4.0%). Thymol methyl ether was earlier identified as a substantial component of *C. cernuum* root EOs (c. 8.4%).

Bourrel et al. [37] studied the antimicrobial activity of EOs from roots of *I. helenium* originating from Central Europe. MIC values determined against *E. coli* (from 2000 μg/mL to >4000 μg/mL), *S. aureus* (62.5–2000 μg/mL), *P. aeruginosa* (≥4000 μg/mL), and *Candida albicans* (62.5–2000 μg/mL) varied depending on the assay method used. Boatto et al. [38] confirmed the activity of the *I. helenium* EOs towards *S. aureus* and *Streptococcus pyogenes* and the low effectiveness of the EO towards Gram-negative bacteria and *Streptococcus faecalis*. The EO from *I. helenium* examined by Deriu and coworkers [39] was active against *Enterococcus faecalis* ATCC 24912 (MIC = 2.9 mg/mL), *S. aureus* ATCC 29213 (MIC = 0.6 mg/mL), *E. coli* ATCC 25922 (MIC = 14.8 mg/mL), *P. aeruginosa* (MIC = 14.8 mg/mL) and *C. albicans* (MIC: 0.009–0.07 mg/mL). Antistaphylococcal activity of the *I. helenium* EO was further investigated by Stojanović-Radić et al. [40] using *S. aureus* ATCC 6538 strain (MIC: 0.01 μL/mL = 13.00 μg/mL). It was shown that the EO increased the bacterial membrane permeability, leading to cell autolysis. The effects on the cell-membrane function may also be engaged in the anticandidal activity of *I. helenium* root EO (MIC: 0.009–0.312 μg/mL) [41]. EOs from roots and aerial parts of *T. speciosa* collected in Bosnia and Herzegovina demonstrated antimicrobial activity against *S. aureus* (MIC: 1.1–15.0 mg/mL), *P. aeruginosa* (MIC: 4.0–11.0 mg/mL), *E. coli* ATCC 35210 (MIC: 1.0–7.0 mg/mL) and *C. albicans* (MIC: 1.0–15.0 mg/mL). The EOs from roots were more active than those distilled from the aerial parts of the plant [33]. The EOs obtained from different organs of *T. speciosa* plants of French origin, cultivated in the Garden of Medicinal Plants, Maj Institute of Pharmacology PAS were active against *S. aureus* ATCC 29213 (MIC: 7.8–31.3 μL/mL) and *E.coli* ATCC 25922 (MIC: 7.8–62.5 μL/mL). The EOs from *T. speciosa* flowers and leaves were more effective against the tested pathogens than the EO from the plant roots [27]. EOs from roots and aerial parts of *C. cernuum* were tested against Gram-positive (*S. aureus* ATCC 29213, *E. faecalis* ATCC 29212) and Gram-negative (*E. coli* ATCC 25922, *Klebsiella pneumoniae* ATCC 700603, *P. aeruginosa* ATCC 27853, *Serratia marcescens* ATCC 13880 and *Acinetobacter baumanii* ATCC 19606) bacteria. The most susceptible of the tested microorganisms was *A. baumanii* (MIC = 11.7 μL/mL) the most resistant turned out to be *S. marcescens* (MIC ≥ 250 μL/mL). Up to the concentrations of 2.08 mg/mL (APEO) and 3.38 mg/mL (REO), the EOs from *C. macrocephalum* did not inhibit the growth of *E. coli* ATCC 25922 and *P. aeruginosa* ATCC 27853 strains. It was expected, taking into consideration the MIC values determined for the EOs from *I. helenium* and *T. speciosa*. Both MRSA-susceptible (ATCC 29213) and MRSA-resistant (ATCC 43300) strains of *S. aureus* were inhibited by the maximum concentrations of EOs used in the assay. The proper assessment of the antimicrobial activity of *C. macrocephalum* EOs should be conducted using higher concentrations of APEO and REO, which could not be performed in the current study due to the limited availability of the EOs.

Cytotoxic activities of the APEO and REO were assessed using a panel of human skin cell lines, including fibroblasts, keratinocytes (HaCaT), and melanoma cells (A375, C32, and SK-MEL-28). The APEO exerted a moderate cytotoxic effect on the melanoma cell lines (IC_50_: 37.9–51.5 nL/mL, after 48 h treatment), but its toxicity against keratinocytes was at the same level (IC_50_ = 48.9 nL/mL). The cytotoxic effect of APEO was less pronounced in human fibroblasts (IC_50_ = 70.6 nL/mL), suggesting some selectivity of action. The activity of REO was stronger but not selective. The IC_50_ values determined for both normal (HaCaT, fibroblasts) and melanoma cell lines, after 48 h treatment with REO, ranged from 3.2 nL/mL (A375 cells) to 7.5 nL/mL (C32 cells). Alantolactone, the dominant component of REO in micromolar concentrations, demonstrated cytotoxic activity against a variety of cancer lines in vitro, including mouse and human melanoma cells [42,43,44]. The sesquiterpene lactone significantly inhibited the proliferative, migratory, and invasive capacity of A375 melanoma cells by inhibiting the Wnt/β-catenin signaling pathway [43]. Moreover, in A375 cells as well as in the triple-negative breast cancer cells MDA-MB-231, alantolactone exerts its antiproliferative and apoptosis-inducing effect by the inhibition of signal transducer and activator of transcription 3 (STAT3) signaling pathway. In contrast to REO, the cytotoxic activity of alantolactone seems to be selective [44]. The compound undoubtedly contributed to the cytotoxic activity of REO, but its effect was modified by the other ingredients of the oil. The EOs distilled from roots and aerial parts of *C. cernuum*, tested against the same cell lines, were less active (IC_50_: 71.7–107.2 nL/mL). Moreover, their cytotoxic effect was not selective, and the fibroblast cell line was relatively strongly affected (IC_50_: 75.7–83.0 nL/mL) [26]. The EO from flowers of *T. speciosa* displayed cytotoxic activity comparable to that of REO (IC_50_: 5.1–17.1 μg/mL, 48 h treatment; cells: A375, C32, HaCaT, and fibroblasts). The highest IC_50_ value was determined for fibroblasts, suggesting some selectivity towards melanoma cells. Cisplatin applied as a reference drug, demonstrated high cytotoxicity against melanoma cells (A375, C32) and keratinocytes (IC_50_: 2.8–3.7 μg/mL) and was significantly less toxic against fibroblasts (IC_50_ > 25 μg/mL) [27].

The results of the apoptosis assay revealed a dose-dependent increase in the percentage of apoptotic cells in the melanoma cell lines treated either with APEO (50, 100, and 200 nL/mL) or with REO (6, 12, and 25 nL/mL), especially marked in A375 and SK-MEL-28 cells. The apoptosis-inducing activity of APEO and REO towards melanoma cells was further supported by the results of Western blotting. In the treated cells, initiator caspase-8 and caspase-9 and executioner caspase-3 and caspase-7 zymogen levels were dose-dependently reduced, except for the C32 cells, where the procaspase-8 was undetectable. The reduction of zymogen levels was accompanied by their cleavage to active forms. Concomitantly, the level of antiapoptotic Mcl-1 protein decreased in the cells treated with the EOs. Previously investigated EO from whole plants of *C. abrotanoides* [35] and EO from *T. speciosa* flowers [27] reduced the viability of human cancer cells in vitro, at least in part, via induction of the apoptotic process.

## 4. Materials and Methods

### 4.1. General Experimental Procedures

GC-MS-FID analyses of essential oils were performed using a Trace GC Ultra Gas Chromatograph coupled with a DSQII mass spectrometer (Thermo Electron, Waltham, MA, USA). Simultaneous GC-FID and GC-MS analyses were performed with an MS-FID splitter (SGE Analytical Science, Ringwood, VIC, Australia). The mass range was set at 33–550 amu, ion source-heating: 200 °C; ionization energy: 70 eV. One microliter of essential oil solution (80% *v*/*v*), diluted in pentane:diethyl ether, was injected in a split mode at the split ratios (50:1). Chromatograph operating conditions were as follows: capillary column Rtx-1 MS (60 m × 0.25 mm i.d., film thickness 0.25 μm), and temperature program: 50 °C (3 min)—300 °C (30 min) at 4 °C/min. Injector and detector temperatures were 280 °C and 300 °C, respectively. Helium was used as a carrier gas (constant pressure: 300 kPa). The relative composition of each essential oil sample was calculated from GC peak areas according to total peak normalization.

### 4.2. Plant Material

Seeds of *Carpesium macrocephalum* Franch. & Sav. were supplied by the Perm State University Botanic Garden in Perm (Russia). The seeds were collected in 2016 from plants growing outdoors in the Botanic Garden (58°00′ N; 56°19′ W) and were sown at the end of March 2020 into multipots with garden soil. In the stage of several mature leaves, the plants were transferred to the pots with a substrate composed of garden soil, peat, and sand. Plants were grown in a glasshouse of the Garden of Medicinal Plants, Maj Institute of Pharmacology PAS in Krakow, under controlled conditions (temperatures by day 18–38 °C; by night 12–18 °C), without any chemical treatment. In the third week of May, the plants were transferred into the open field. Aerial parts and roots of the plants were collected at the second year of growth, at the beginning of the flowering period (July), and dried under shade at room temperature. The voucher specimen (6/22) was deposited in the collection kept at the Garden of Medicinal Plants, Maj Institute of Pharmacology PAS, Kraków, Poland. The dry plant material was stored no longer than six months before analysis.

### 4.3. Isolation of Essential Oil

EOs from the dried aerial parts (leaves, stalks, flowers; 2.7 kg) or roots (0.7 kg) of *C. macrocephalum* were obtained by hydrodistillation in a Clevenger-type apparatus. Each process lasted 5 h, and a portion of 100–550 g of the dry plant material was used once. The EO from aerial parts (APEO, translucent colorless liquid) and roots (REO, yellowish liquid) were dried over anhydrous magnesium sulfate and stored at 4 °C in the dark until tested and analyzed.

### 4.4. Identification of Essential Oil Constituents

Volatiles from the essential oils were identified based on their MS spectra and their comparison with those from mass spectra libraries: NIST 2012, Wiley Registry of Mass Spectral Data 8th edition and MassFinder 4.1, along with the relative retention indices (RI) on DB-1 column (available from MassFinder 4.1) and on HP-5ms column (available from NIST 2012 or [45]). The identification of isoalantodiene was supported by literature data [46,47,48].

### 4.5. Antibacterial Activity of Carpesium macrocephalum Essential Oils

#### 4.5.1. Bacterial Strains and Culture Conditions

Four reference strains were used in the current study: *Staphylococcus aureus* ATCC 29213 (methicillin-susceptible *S. aureus*—MSSA), *S. aureus* ATCC 43300 (methicillin-resistant *S. aureus*—MRSA), *Escherichia coli* ATCC 8739, and *Pseudomonas aeruginosa* ATCC 27853. The bacteria were cultured on Columbia agar with 5% sheep blood (bioMeriéux, Warsaw, Poland) and incubated for 18 h at 37 °C in an aerobic atmosphere prior to each experiment.

#### 4.5.2. Determination of the Minimum Inhibitory Concentrations (MICs) and the Effectiveness of the Tested *C. macrocephalum* EOs against the Reference Bacterial Strains

The MICs of the essential oils (APEO and REO) against the selected Gram-positive (*S. aureus*) and Gram-negative (*E. coli*, *P. aeruginosa*) bacteria were determined by the serial microdilution method in Mueller-Hinton broth (MHB; Sigma-Aldrich, Darmstadt, Germany) according to the Clinical and Laboratory Standards Institute recommendations (CLSI, 2012) [49]. Thymol (Sigma-Aldrich, Darmstadt, Germany) and gentamycin sulfate (solution for injections; Krka, Novo Mesto, Slovenia) were used as positive controls. Thymol, a monoterpenoid being found in numerous essential oils and having well-documented antimicrobial activity [50] was chosen as a reference in the previously described experiments [26,27]. The antibacterial activity of this compound against *Staphylococcus aureus* ATCC 43300 was better than those of β-lactam antibiotics tested in the same experiment [51]. Briefly, 50 µL of the appropriate concentration of EOs or the compound of known antibacterial activity (thymol, gentamicin sulfate) was added to a 96-well microplate. Concentrations of APEO in a range from 1.02 µg/mL to 2080 µg/mL and REO in a range from 1.65 µg/mL to 3380 µg/mL were prepared by dissolving the substances in Tween 80 (1.0%, *v*/*v*) (Sigma-Aldrich, Darmstadt, Germany) and diluting by MHB. The solutions of thymol (from 625 µg/mL to 1.22 µg/mL) were prepared by dissolving the compound in dimethyl sulfoxide (2.0%, *v*/*v*) (DMSO, Loba Chemie, Mumbai, India) and diluting it using MHB. In turn, concentrations of gentamycin sulfate, in a range from 0.31 µg/mL to 625 µg/mL, were prepared by diluting the antibiotic solution using MHB. In the next step, 50 µL of a bacterial suspension at 10^6^ CFU/mL was transferred to each well of the microplate. After an 18 h incubation at 37 °C, MICs for individual substances were determined by adding 20 µL resazurin solution (0.02%, *w*/*v*; Sigma-Aldrich, Darmstadt, Germany) to the microwells. The color change from dark blue to pink after a 3 h incubation at 37 °C indicated the presence of live bacteria. The first well in which the blue color persisted determined the MIC value. The bacteria suspension with 1.0% (*v*/*v*) Tween 80 or 2.0% (*w*/*v*) DMSO was regarded as a negative control. All tests were run in duplicate.

### 4.6. Cytotoxic Activity of Essential Oils from Carpesium macrocephalum

#### 4.6.1. Cell Lines and Culture Conditions

Human melanoma cell lines A375, C32, and SK-MEL-28 and dermal fibroblasts CCD25Sk were purchased from the American Type Culture Collection (ATCC, Manassas, VA, USA). Skin keratinocytes HaCaT were obtained from AddexBio (San Diego, CA, USA). Melanoma cells A375 and C32, fibroblasts, and keratinocytes were cultured in Dulbecco’s Modified Eagle’s Medium, while SK-MEL-28 cells were maintained in Eagle’s Minimum Essential Medium. Both growth media were supplemented with 10% fetal bovine serum, 100 U/mL penicillin, and 100 μg/mL streptomycin. The media and supplements were purchased from Thermo Fischer Scientific (Waltham, MA, USA). Cells were incubated at 37 °C in a humidified atmosphere of 5% CO_2_.

#### 4.6.2. Cell-Viability Assay

The examined cells were seeded in 96-well plates at a density of 1 × 10^4^ cells per well and allowed to adhere for 24 h. APEO and REO were solubilized in dimethyl sulfoxide (DMSO) at a ratio of 1:10 (*v*/*v*) and mixed with the culture medium to obtain final concentrations of 12.5, 25, 50, 100, and 200 nL/mL (APEO) or 3.1, 6.2, 12.5, 25, and 50 nL/mL (REO). The cells were then treated either with the solutions of EOs or with the culture medium containing DMSO (0.2%, control for the APEO-treated group; 0.05%, control for the REO-treated group). The viability of the cells was evaluated after 24 h and 48 h of the treatment by adding 3-(4,5-dimethyl-2-thiazolyl)-2,5-diphenyl-2H-tetrazolium bromide (MTT) solution to each well and subsequent incubation of the cells at 37 °C for 2 h. Then, the cells were lysed in DMSO supplemented with 1% (*v*/*v*) Sorensen’s glycine buffer. The absorbance was measured at 570 nm. The half-maximal inhibitory concentration values (IC_50_) were calculated using GraphPad Prism 7 software.

#### 4.6.3. Apoptosis Assay

Cells were seeded at a density of 1 × 10^5^ cells per well in 6-well plates and allowed to adhere for 24 h. Then, cells were treated with the respective concentrations of APEO and REO for 48 h. Control cells were treated with 0.2% DMSO. Floating and adherent cells were collected and assayed using a Dead Cell Apoptosis Kit with annexin V-fluorescein isothiocyanate (FITC) and propidium iodide (PI) for flow cytometry (#V13242, Thermo Fisher Scientific, Waltham, MA, USA), according to the manufacturer’s protocol. Briefly, cells were resuspended in 100 μL annexin-binding buffer containing 5 μL annexin V-FITC conjugate solution and 1 μg/mL PI followed by 15 min incubation at room temperature. Subsequently, 400 μL of the annexin-binding buffer was added, and the cells were quantified with a DxFLEX flow cytometer (Beckman Coulter, Brea, CA, USA).

#### 4.6.4. Western Immunoblot

Cells were treated with the respective concentration of APEO, REO, or with 0.2% DMSO (control) for 48 h, then adherent and floating cells were harvested and sonicated. The Lowry assay was performed to quantify protein content in the obtained homogenates. Proteins (20–40 μg) were resolved on 7.5%, 10%, or 12% SDS-PAGE gels using the Mini-Protean Tetra system (Bio-Rad, Hercules, CA, USA). Proteins were transferred to nitrocellulose membranes (Bio-Rad) using the Mini Trans-Blot Cell wet blotting system (Bio-Rad). Membranes were blocked with 5% skim milk for 1 h at room temperature and probed overnight at 4 °C with primary antibodies. The following antibodies purchased from Cell Signaling Technology (Danvers, MA, USA) were used: caspase-8 (#9746, 1:1000), caspase-9 (#9508, 1:1000), Mcl-1 (#5453, 1:1000), caspase-3 (#9662, 1:1000), caspase-7 (#12827, 1:1000), PARP (#9542, 1:1000). Antibody against actin (#A2066, 1:2000) was obtained from Sigma-Aldrich (Saint Louis, MO, USA). After extensive washes, a secondary antibody solution in 5% skim milk (anti-mouse IgG-HRP, Sigma-Aldrich, #A9044, 1:5000 or anti-rabbit IgG-HRP, Sigma-Aldrich, #A9169, 1:5000) was added for 1 h at room temperature. Membranes were incubated with ECL-HRP substrate (GE Healthcare, Chicago, IL, USA), and the signal was detected using the Alliance Q9 Advanced imaging system (Uvitec, Cambridge, UK).

#### 4.6.5. Statistical Analysis

Data were analyzed in GraphPad Prism 7 software using a one-way ANOVA followed by Tukey’s test and reported as mean ± standard deviation. Values of *p* < 0.05 were considered as statistically significant.

## 5. Conclusions

The EOs distilled from roots and aerial parts of *C. macrocephalum* share some similarities in composition with the EOs found in taxonomically related species. The high content of eudesmanolides in REO reflects the relationships with *Telekia* and *Inula* spp. and the results of the previous phytochemical studies on the plant. Moreover, the presence of eudesmanolides in REO seems to be responsible for the high cytotoxic activity of the oil. Its cytotoxic effect on keratinocytes and human skin fibroblasts should be taken into consideration before the external application of the plant extract as a component of medicines or cosmetics. EOs from *I. helenium* (rich in eudesmanolides) demonstrated good anticandidal and antistaphylococcal activity. The antimicrobial activity of REO and APEO should be further investigated using a wider concentration range and including additional bacterial strains as well as yeast-like fungi.

## Figures and Tables

**Table 1:** Chemical composition of *Carpesium macrocephalum* essential oils distilled from aerial parts (APEO) and roots (REO) of the plants cultivated in the open field.

No	Compound	APEO	REO	RI Exp. ^1^	RI Lit. ^2^	Identification Method
		Amount [%]				
1	2-Pentylfuran	tr	0.1	965	971	RI, MS
2	Octanal	0.5	0.1	975	982	RI, MS
3	*β*-Myrcene		tr	976	987	RI, MS
4	(E)-2-(2-Pentenyl)furan	2.0		977	983	RI, MS
5	Benzeneacetaldehyde		tr	1007	1009	RI, MS
6	*p*-Cymene		tr	1008	1013	RI, MS
7	Limonene	0.1	tr	1018	1025	RI, MS
8	(*Z*)-*β*-Ocimene	tr	tr	1036	1029	RI, MS
9	(*E*)-Oct-2-en-1-al		tr	1037	1034	RI, MS
10	(*E*)-*β*-Ocimene	tr		1039	1041	RI, MS
11	(3*E*,5*E*)-3,5-Octadien-2-one	tr	tr	1048	1057	RI, MS
12	*trans*-Linalooloxide (furanoid)	0.1	tr	1056	1058	RI, MS
13	Non-1-en-3-ol	tr	tr	1065	1068	RI, MS
14	*p*-Cymenene		tr	1074	1076	RI, MS
15	6-Methyl-3*E*,5-heptadien-2-one		tr	1076	1076	RI, MS
16	Terpinolene	0.1	tr	1077	1078	RI, MS
17	Nonanal	0.2		1081	1084	RI, MS
18	**Linalool**	**9.3**	**0.1**	**1085**	**1081**	**RI, MS**
19	Limona ketone	tr		1096	1105	RI, MS
20	Non-3-en-2-one	tr	tr	1113	1114	RI, MS
21	(2*E*,6Z)-Nonadienal	tr		1122	1125	RI, MS
22	(*E*)-Non-2-enal	0.1	tr	1135	1233	RI, MS
23	**Nerol oxide**	**3.6**	**0.1**	**1137**	**1137**	**RI, MS**
24	*p*-Ethylbenzaldehyde	tr		1142	1147	RI, MS
25	4-(2-Methyl-1-cyclohex-1-yn)but-2*E*-enal	0.2	2.2	1150	1149	RI, MS
26	Cymen-9-ol	0.1	tr	1161	1157	RI, MS
27	Terpinen-4-ol	tr	tr	1161	1161	RI, MS
28	Caprylic acid	0.1		1168	1167	RI, MS
29	*α*-Terpineol	2.2	0.1	1172	1174	RI, MS
30	*p*-Cumic aldehyde	0.1	0.2	1194	1214	RI, MS
31	*β*-Cyclocitral	tr		1201	1197	RI, MS
32	Nerol		1.0	1210	1215	RI, MS
33	**Thymol methyl ether**		**7.1**	**1214**	**1215**	**RI, MS**
32 + 33	**Nerol + Thymol methyl ether (0.9:0.1)**	**26.0**		**1215**	**1215**	**RI, MS**
34	Geraniol	0.9	0.1	1234	1232	RI, MS
35	Geranial	0.4	tr	1242	1244	RI, MS
36	*p*-tert-Butylphenol	tr	tr	1246	1250	RI, MS
37	*α*-Ionene	tr	tr	1252	1255	RI, MS
38	Pelargonic acid	tr	0.2	1255	1260	RI, MS
39	Isoprop-1-enyl-2-isopropylbenzene		0.2	1138	1140	RI, MS
40	Thymol	0.1	0.1	1267	1260	RI, MS
41	Deca-2,4-dienal	tr	tr	1272	1270	RI, MS
42	Carvacrol	tr	0.1	1308	1278	RI, MS
43	7*α*-Silphiperfol-5-ene	tr	0.1	1326	1329	RI, MS
44	Eugenol	tr		1329	1331	RI, MS
45	Dehydro-*ar*-ionene	0.1		1330	1332	RI, MS
46	Presilphiperfol-7-ene		0.2	1336	1333	RI, MS
47	1,1,4,5-Tetramethylindane	tr		1341	1355	RI, MS
48	7β-Silphiperfol-5-ene	0.2	0.6	1345	1350	RI, MS
49	*α*-Longipinene	tr	0.1	1352	1358	RI, MS
50	Silphiperfol-1-ene	0.1	0.1	1360	1358	RI, MS
51	(*E*)-*β*-Damascenone	tr		1361	1361	RI, MS
52	Longicyclene	0.1	tr	1371	1376	RI, MS
53	*α*-Copaene	0.3		1373	1377	RI, MS
54	Silphiperfol-6-ene		0.3	1375	1378	RI, MS
55	**Modheph-2-ene**	**1.7**	**4.6**	**1381**	**1382**	**RI, MS**
56	*β*-Bourbonene	0.3		1383	1386	RI, MS
57	*α*-Isocomene	0.8	1.8	1387	1388	RI, MS
58	**2,5-Dimethoxy-*p*-cymene** **(Thymohydroquinone dimethyl ether)**	**7.9**	**6.6**	**1399**	**1399**	**RI, MS**
59	Italicene	0.2	tr	1403	1404	RI, MS
60	*β*-Isocomene	0.7	1.9	1406	1407	RI, MS
61	Isobornyl isobutyrate	0.6		1410	1410	RI, MS
62	7,8-Dihydro-*β*-ionone	tr	tr	1412	1411	RI, MS
63	(*E*)-*β*-Caryophyllene	0.3	0.4	1417	1421	RI, MS
64	Geranylacetone	0.3	0.1	1427	1428	RI, MS
65	*trans*-*α*-Bergamotene	0.1	0.1	1432	1434	RI, MS
66	*epi*-*β*-Santalene	0.2	0.3	1442	1446	RI, MS
67	*α*-Himachalene	tr	0.1	1445	1447	RI, MS
68	*α*-Humulene	tr	0.1	1451	1455	RI, MS
69	*β*-Santalene	0.1	tr	1454	1459	RI, MS
70	8,9-Didehydrothymyl isobutyrate	tr	0.3	1456	1458	RI, MS
71	Thymyl isobutyrate		0.4	1457	1462	RI, MS
72	*allo*-Aromadendrene	0.6		1458	1462	RI, MS
73	(*E*)-*β*-Ionone	0.4	0.1	1462	1468	RI, MS
74	6-Isopropenyl-4,8*α*-dimethyl-1,2,3,5,6,7,8,8*α*-octahydro-2-naphthalenyl acetate	0.1	tr	1466		MS
75	*cis*-*β*-Guaiene		tr	1468	1469	RI, MS
**76**	**Neryl isobutyrate**	**12.1**	**2.4**	**1471**	**1469**	**RI, MS**
77	*γ*-Himachalene	tr	tr	1474	1479	RI, MS
78	Thujopsadiene	0.2	tr	1478	1470	RI, MS
79	Patchoulene	0.6	0.2	1482	1473	RI, MS
80	1,4-Dioxaspiro [4,6]undec-6-yl acetate	0.1	0.8	1486		MS
81	Dibenzofuran	tr	tr	1490	1504	RI, MS
82	Bicyclogermacrene		0.2	1491	1494	RI, MS
83	*α*-Selinene	1.2	tr	1492	1495	RI, MS
84	*β*-Bisabolene	0.1	0.2	1500	1500	RI, MS
85	**Isoshyobunone**	**0.9**	**5.6**	**1507**	**1518**	**RI, MS**
86	*δ*-Cadinene	0.2	0.1	1514	1520	RI, MS
87	*cis*/*trans*-Calamenene	tr	0.2	1520	1523	RI, MS
88	10-*epi*-Italicene ether	0.1	tr	1522	1531	RI, MS
89	(*E*)-*β*-Caryophyllene oxide	tr	0.1	1542	1546	RI, MS
90	(*Z*)-Nerolidol	0.1	0.2	1546	1546	RI, MS
91	(*E*)-Nerolidol	1.5	1.1	1556	1555	RI, MS
92	Neryl isovalerate	0.4	0.3	1562	1565	RI, MS
93	**Caryophyllene epoxide**	**0.5**	**4.0**	**1570**	**1571**	**RI, MS**
94	Unidentified (MS: 148/133/91/187 M220)	0.1	0.4	1579		
95	Isoaromadendrene epoxide	0.2	1.1	1582	1592	RI, MS
96	*β*-Himachalene epoxide	0.1	1.3	1594	1594	RI, MS
97	Unidentified (MS: 162/147/120/173 M206)	0.4		1597		
98	Unidentified (MS: 162/147/120/173 M206)	1.2	0.2	1600		
99	Zierone	0.1	0.8	1611	1582 ^3^	RI, MS
100	Torryeol		tr	1612	1607	RI, MS
101	2,5-Ditert-butylbenzo-1,4-quinone	0.2	0.2	1613	1613	RI, MS
102	Cubenol	tr	0.4	1619	1620	RI, MS
103	Caryophylla-3(15),7(14)-dien-6-ol	0.1	0.3	1625	1630	RI, MS
104	*T*-Muurolol	1.0	1.5	1631	1633	RI, MS
105	*β*-Selinenol	0.3	1.4	1635	1638	RI, MS
106	*α*-Cadinol	0.2	0.4	1638	1643	RI, MS
107	Eudesm-4(15)-en-7-ol	tr	0.7	1640	1643	RI, MS
108	Unidentified (MS: 162/147/91/119/41)	tr	0.1	1649		
109	6-Methoxythymyl isobutyrate	1.7	1.5	1656	1659	RI, MS
110	*α*-Acorenol	0.1	0.2	1667	1667	RI, MS
111	2,3-Dihydrofarnesol	0.1	0.2	1670	1674	RI, MS
112	6-Methoxy-8,9-didehydrothymyl isobutyrate	tr	0.1	1676	1676	RI, MS
113	10-Isobutyryloxy-8,9-didehydrothymol methyl ether	tr	tr	1684	1684	RI, MS
114	*β*-(*E*)-Santalol	tr	tr	1684	1680	RI, MS
115	n-Pentadecanal	0.4		1692	1702	RI, MS
116	Phenantrene	0.1		1747	1740	RI, MS
117	*β*-Santalol acetate	0.2	tr	1801	1800	RI, MS
118	Unidentified (MS: 71/119/134/43 M223)	0.5		1818		
119	Diisobutyl phthalate (artifact)	2.9	1.5	1826	1826	RI, MS
120	Dihydroisoalantolactone	0.2		1860	1875	RI, MS
121	**Alantolactone**	**0.1**	**29.3**	**1875**	**1873**	**RI, MS**
122	9-Isobutyryloxythymyl isobutyrate	0.1	1.1	1878	1884	RI, MS
123	10-Isobutyryloxy-8,9-didehydrothymyl isobutyrate	0.1	0.8	1890	1887	RI, MS
124	(5*E*,9*E*)-Farnesylacetone	tr	0.4	1895	1885	RI, MS
125	Isoalantolactone	0.7	0.7	1900	1912	RI, MS
126	**Alloalantolactone (1-Deoxyivangustin/(+)-Diplophyllin)**		**4.4**	**1914**	**1916 ^3^**	**RI, MS**
127	Dibutyl phthalate (artifact)	0.4	tr	1920	1912	RI, MS
128	Isoalantodiene (3-Dehydroalantolactone)		0.1	1922		MS
129	Unidentified (MS: 121/91/79/95/119/145/190/105/41 M232)		0.6	1931		
130	Palmitic acid	0.2	0.2	1940	1952	RI, MS
131	9-(2-Methylbutyryloxy)thymyl isobutyrate		0.2	1964	1964	RI, MS
132	10-Isobutyryloxy-8,9-epoxythymyl isobutyrate	tr	2.2	1985	1972	RI, MS
133	**(*E*,*E*)-Geranyl linalool**	**6.5**	**tr**	**2012**	**2020**	**RI, MS**
134	10-(2-methylbutyryloxy)-8,9-epoxythymyl isobutyrate	0.4	0.3	2068	2084	RI, MS
135	Tricosane	0.2		2293	2300	RI, MS
136	Tetracosane	tr		2394	2400	RI, MS
137	Heptacosane	0.2	0.1	2659	2700	RI, MS
	**Sum of identified**	**96.0**	**96.7**			
	**Yield of the essential oil [%]**	**0.08**	**0.13**			

^1^ RI_exp_.—experimental retention index; ^2^ RI_lit_.—literature retention index calculated on DB-1 column; ^3^ RI_lit_—calculated on HP-5ms column; tr—<0.05% (trace).

**Table 2:** Antibacterial activities of essential oils from aerial parts (APEO) and roots (REO) of *Carpesium macrocephalum*.

Tested Chemicals	Minimum Inhibitory Concentration (µg/mL)			
	*Staphylococcus aureus* ATCC 29213 (MSSA)	*Staphylococcus aureus* ATCC 43300 (MRSA)	*Escherichia coli* ATCC 25922	*Pseudomonas aeruginosa* ATCC 27853
APEO	2080.0 ± 0.0	2080.0 ± 0.0	nd	nd
REO	3380.0 ± 0.0	3380.0 ± 0.0	nd	nd
Thymol ^a^	0.9 ± 0.0	0.9 ± 0.0	7.5 ± 0.0	7.5 ± 0.0
Gentamycin sulphate	0.31 ± 0.0	19.5 ±0.0	0.61 ± 0.0	1.22 ± 0.0

^a^ 1 μg/mL of thymol corresponds to c. 1.04 nL/mL (weighted at the melting point of thymol); nd—not determined.

## Data Availability

The raw data that support the findings of this study are available from the authors, [A.W.-B., Ł.S., P.K., A.S.], upon reasonable request.
